# The increasing menace of dengue in Guangzhou, 2001–2016: the most important epicenter in mainland China

**DOI:** 10.1186/s12879-019-4504-3

**Published:** 2019-11-27

**Authors:** Zhoubin Zhang, Qinlong Jing, Zongqiu Chen, Tiegang Li, Liyun Jiang, Yilan Li, Lei Luo, John Marshall, Zhicong Yang

**Affiliations:** 1 Guangzhou Center for Disease Control and Prevention, Guangzhou, 510440 People’s Republic of China; 2 University of California, Berkeley, USA

**Keywords:** Dengue fever, Epidemiology, Vector surveillance

## Abstract

**Background:**

Dengue is the most prevalent mosquito-borne disease in the world, with China affected seriously in recent years. 65.8% of dengue cases identified in mainland China since 2005 were reported from the city of Guangzhou.

**Methods:**

In this study, we described the incidence rate and distribution of dengue cases using data collected form National Notifiable Infectious Disease Reporting Information System data in Guangzhou for 2001 to 2016. All dengue cases were investigated using standardized questionnaire.

**Results:**

A total of 42,469 dengue cases were reported, with an average annual incidence rate of 20.99 per 100,000 resident population. Over this time period, the incidence rate of indigenous cases increased. Dengue affected areas also expanded sharply geographically from 58.1% of communities affected during 2001–2005 to 96.4% of communities affected in 2011–2016. Overall 95.30% of the overseas imported cases were reported during March and December, while 99.79% of indigenous cases were reported during July and November. All four dengue virus serotypes were identified both in imported cases and indigenous cases. The *Aedes albopictus* mosquito was the only vector for dengue transmission in the area.

**Conclusions:**

Guangzhou has become the dengue epicenter in mainland China. Control strategies for dengue should be adjusted to the epidemiological characteristics above and intensive study need to be conducted to explore the factors that driving the rapid increase of dengue.

## Background

Dengue is the most prevalent mosquito-borne disease in human beings, with estimated 390 million infections and 96 million symptomatic cases in the endemic areas, which has a population of 3.6 billion [1]. The pathogen, dengue virus (DENV), consists of four antigenically related and evolutionarily distinct serotypes (DENV-1, DENV-2, DENV-3, and DENV-4), which are mainly transmitted by *Aedes aegypti* and *Aedes albopictus*. The clinical spectrum of dengue ranges from asymptomatic infection to severe disease, which can progress to highly fatality dengue hemorrhagic fever (DHF) and dengue shock syndrome (DSS). A subsequent infection with different serotypes may result in more severe dengue due to a phenomenon called the antibody dependent enhancement (ADE). Dengue has evolved from a sporadic disease to an rapidly spreading and severe global public health problem [2]. More than 70% of the population at risk lives in Asian Pacific region, where is deemed as the epicenter of dengue transmission [1]. A vaccine for dengue is now available but its efficacy in the population is still under further investigation.

China has experienced local dengue transmission mainly in the southern provinces such as Hainan, Guangdong, Guangxi, Fujian, Zhejiang since 1978, which was the beginning year of the dengue epidemic in mainland china [3, 4]. From 1978 to present, dengue fever has occurred every year in Guangdong Province, China, and most of the cases are from Guangzhou, the capital city of Guangdong Province. Guangzhou located at 112°57 E to 114°3 E and 22°26 N to 23°56 N, with 10 administrative districts and 2 satellite cities, covering 7434.40 km^2^ and with a current population of more than 14.04 million. Guangzhou is one of largest city in the world, having a population density of 1889 people per square kilometer. The climate in Guangzhou is a humid subtropical climate influenced by the Asian monsoon season. In the past three decades, *Aedes albopictus* had been monitored as the vector for dengue transmission in Guangzhou, with.

no *Aedes aegypti* identified. It ranked second highest in proportion amongst all adult mosquitoes from surveillance at about 7% compared to 8% of Culex fatigans which took the largest proportion. From 1990s, the exchange of commerce and population of Guangzhou has been continuing to increase and the urbanization process and the lack of urban management(a large number of labor population living in slum area with poor daily living environment) had led to the continued widespread of mosquito-borne breeding habitats. After 2005, Guangzhou graduallybecame the epicenter for dengue transmission in china, reporting about 69.0% of the total indigenous cases reported in mainland China and keep disseminating cases to other provinces during dengue season.

In this study, we described the epidemiological characteristics for both locally transmitted and imported dengue cases, virus isolation and vector surveillance in Guangzhou in the past 16 years from 2001 to 2016, to identify transmission trend and seasonality for planning further strategy for dengue control and prevention.

## Methods

### Data collection

According to the Law on Prevention and Treatment of Infectious Diseases of China, suspected or confirmed dengue cases identified in medical institutions, must be reported to the National Notifiable Infectious Disease Reporting Information System (NIDRIS) within 24 h. From 1 September 1989 to 2004, dengue cases were reported by telephone and mail. Since 2004, dengue cases were reported through web-based online system [4]. Once a dengue case was reported, a face-to-face case investigation was then conducted by the municipal and district level of Center for Disease Control and Prevention in Guangzhou within 24 h [5]. The case investigation collected data include age, gender, address, diagnosis type, onset date, probable risk factors and source of infection; a blood sample was also collected for further laboratory confirmation. The cases are classified as either imported or indigenous based on the patients’ travel history and the incubation period (3–14 days) of dengue virus infection.

Monthly mosquito vector surveillance in Guangzhou was conducted by GZCDC, to determine the indices of the *Breteau Index* (BI), the *Standard Space Index* (SSI) and the *Adult Mosquito Density Index* (ADI). The BI measured indoors and SSI measured outdoors are two conventional *Aedes* larval indices applied to evaluate mosquito density [6]. The indices are calculated as follows: BI = number of positive containers per 100 houses, SSI = number of positive containers per 100 outdoor standard spaces (15 square meters). The ADI measurement of adult mosquito was calculated by the number of *Aedes* collected per hour and per person by hand held mosquito capturing device.

### Case definition

According to the Diagnostic Criteria for Dengue Fever (WS216–2008) enacted by the Chinese Ministry of Health, a suspected case was defined as a patient presenting with acute onset of fever (39–40 °C within 24–36 h), and other typical symptoms such as headache, arthralgia, myalgia, malaise, and rash, sometimes accompanied by facial flushing, skin erythema, conjunctival congestion, and leukocytopenia, thrombocytopenia, or a positive tourniquet test. Clinically diagnosed case was defined as a suspected case combined with testing positive for DENV IgM/IgG or NS1 antigen by immune colloidal gold technique in serum. Laboratory confirmed cases was defined as a clinically diagnosed case with a positive DENV RNA detected by real-time fluorescent quantitative reverse transcription-PCR (qRT-PCR), or virus isolation, or a four-fold increase of IgG titer in paired serum samples by Capture ELISA. Cases initially reported as suspected case will be updated to clinically diagnosed case or laboratory confirmed case if a further laboratory test (DENV IgM/IgG or NS1 antigen or qRT-PCR) was positvie within 3 weeks. If a further laboratory test (DENV IgM/IgG or NS1 antigen or qRT-PCR) was negative, a suspected case will be removed from the NIDRIS.

According tothe Diagnostic Criteria for Dengue Fever (WS216–2008) enacted by the Chinese Ministry of Health, all the cases were divided into two groups: uncomplicated cases and severe case. Severe case was defined as a clinically diagnosed case or laboratory confirmed case present the following symptoms: a) severe bleeding: subcutaneous hematoma, gross hematuria, bleeding in the digestive tract, chest and abdomen, vagina, intracranial or other parts. b) Severe organ damage: acute myocarditis, acute respiratory distress syndrome, acute liver injury, acute renal insufficiency, central nervous system damage etc. c) Shock: tachycardia, prolonged capillary filling time > 3 s, weak or undetectable pulse, undetectable blood pressure.

### Data analysis

The study analyzed all case data for Guangzhou City reported from 1 January 2001 to 31 December 2016. The incidence rate was calculated as the reported cases divided by the population at each year-end, which was obtained from the statistics year book from Guangzhou Bureau of Statistics (http://www.gzstats.gov.cn/). To analyze the seasonality of dengue in Guangzhou, we plotted heat maps both for indigenous cases and imported cases by month in each years of the study period. All data were analyzed by R statistical software (version 3.3.3, R Foundation for Statistical Computing, Vienna, Austria), including plotting graphs, heat maps and statistical analysis [7].

## Results

### Overall incidence

During the 16 year period from 2001 to 2016, a total of 42,469 cases were reported to NIDRIS. There were 17,979 laboratory confirmed cases and 24,490 clinically diagnosed cases among these cases, with 15,960 (37.58%) hospitalized cases, 386 severe cases (0.91%) and 5 deaths. The annual average incidence rate was 21.0 cases per 100,000 resident population. Dengue cases for Guangzhou accounted for 65.8% (40,177/61024) of all dengue cases reported in mainland China between 2005 to 2016. There were large dengue outbreaks in consecutive years from 2013 to 2014 with a total of 39,361 cases reported. The biggest dengue outbreak reported in 2014, resulting in 38,029 cases and accounting for 91.92% of all cases reported in Guangzhou during the study period. Furthermore, 14,055 hospitalized cases, 308 severe cases and 5 fatal cases reported in 2014, the first time death occurred since 1990.

### Indigenous cases

**Fig. 1:**
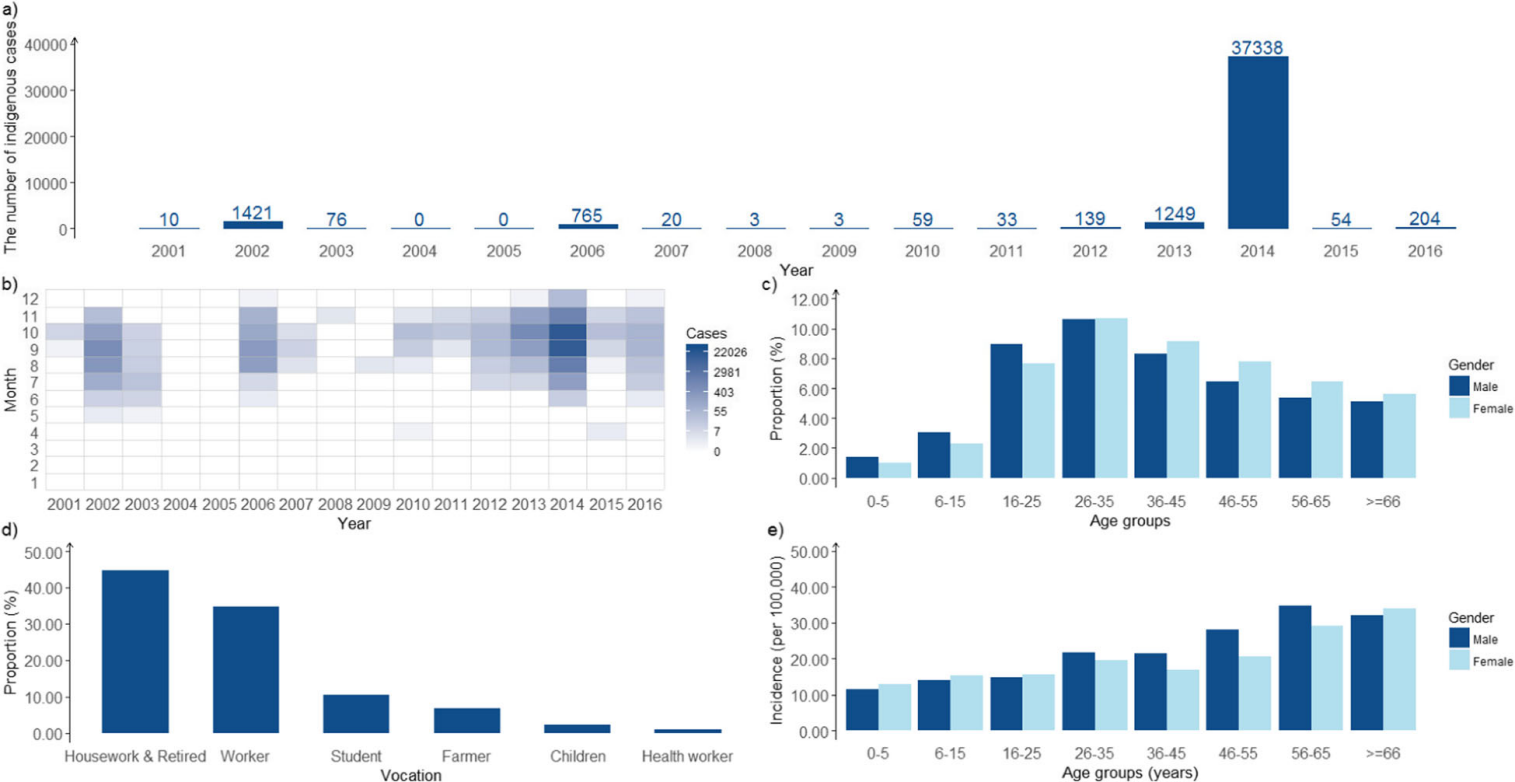
The epidemiological distribution of indigenous cases in Guangzhou, 2001–2016. **a** Yearly distribution of indigenous case; **b** Monthly based heat map for the number of indigenous cases with seasonality; **c** Proportion of total cases in different age groups; **d** Occupation distribution in indigenous cases; **e** Incidence rate of different age groups

A total of 41,374 (97.42%) indigenous cases were reported in the study period, with 5 deaths in 2014 (Fig. 1a). The median incidence rate was 0.43 per 100,000 population (IQR: 0.11–3.01 cases per 100,000), with the highest incidence rate of 285.45 per 100,000 population (37,338 cases) occurred in 2014.

In terms of seasonality, all the indigenous cases were reported between April and December (Fig. 1b), and 99.8% of indigenous cases reported between July and November. Cases peaked in October (48.1%). Only three indigenous cases were reported in April, one in 2010 and two in 2015, however, no following case was reported in the following Mayin those 2 years. No indigenous case were reported in 2004 and 2005. No indigenous cases were reported from January to March in any year during the study period.

**Fig. 2:**
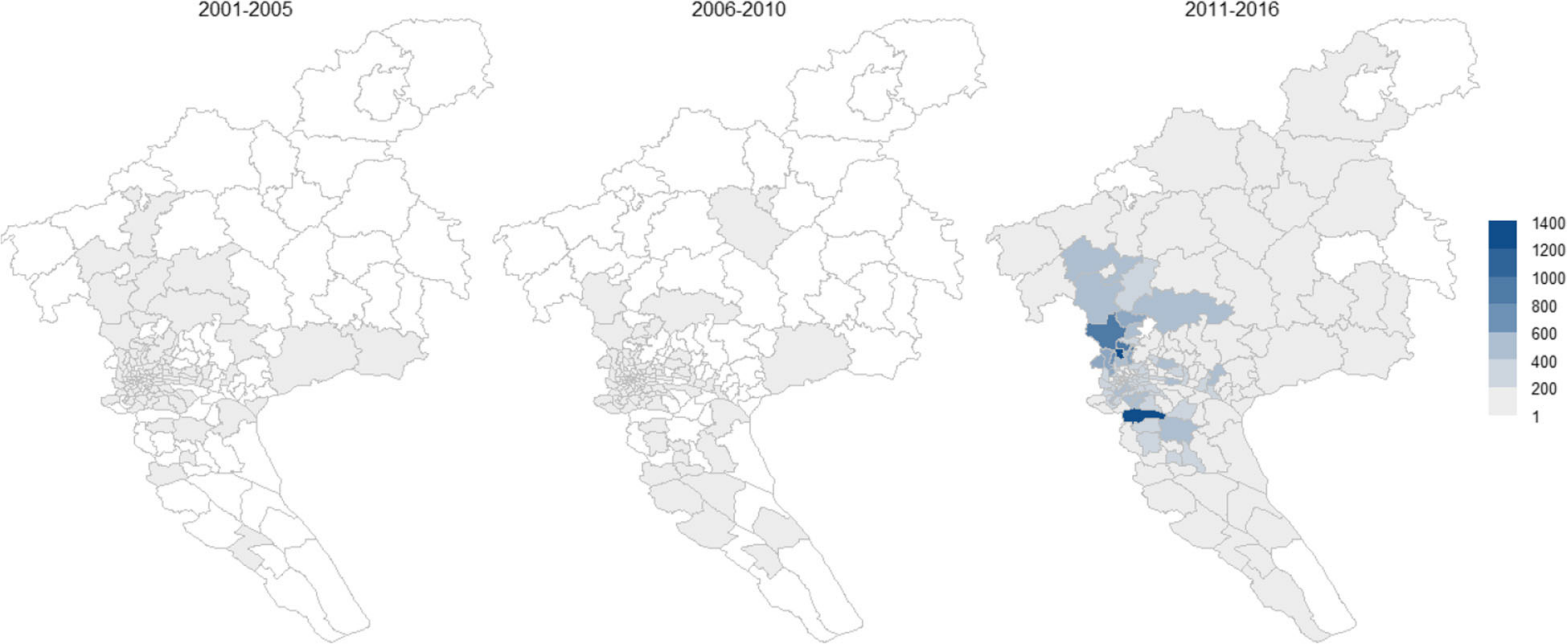
The community-based geographic distribution of indigenous cases in Guangzhou, 2001–2016. The white color community denotes no cases reported

During 2001 to 2016, 161 (96.4%) communities in Guangzhou reported indigenous cases, with most of cases distributed in urban areas. Only 6 communities that located in border region (Timian, Longxue, Zhengguo, Xiaolou, Lvtian and Liuxihe) did not reported cases in the study period (Fig. 2). During 2001–2005, 1507 indigenous cases were reported in 97 (58.1%) communities, while 850 cases in 95 (56.89%) communities were reported during 2006–2010, in which period 23 newly emerging communities were affected by indigenous cases. Between 2011 and 2016, the number of indigenous cases increased dramatically to 38,791 and the number of communities that reported indigenous cases rapidly expanded to 161 (96.41%).

The male-to-female case ratio was 1:1.02 (20,980/21,489) with no gender predominance among indigenous cases during 2001–2016. Age-specific incidence increased by age group, with the highest incidence rate occur in group 56–65 years (31.2 per 100,000). In contrast, the greatest proportion of cases occurred in young adults (16–45), with the highest proportion observed for individuals age from 26 to 35(21.30%). **(**Fig. 1c and e**)**. As for occupation distribution, the housework and the retired population had the largest proportion of 44.8% and followed by adult worker of 34.7% (Fig. 1d).

### Imported cases

**Fig. 3:**
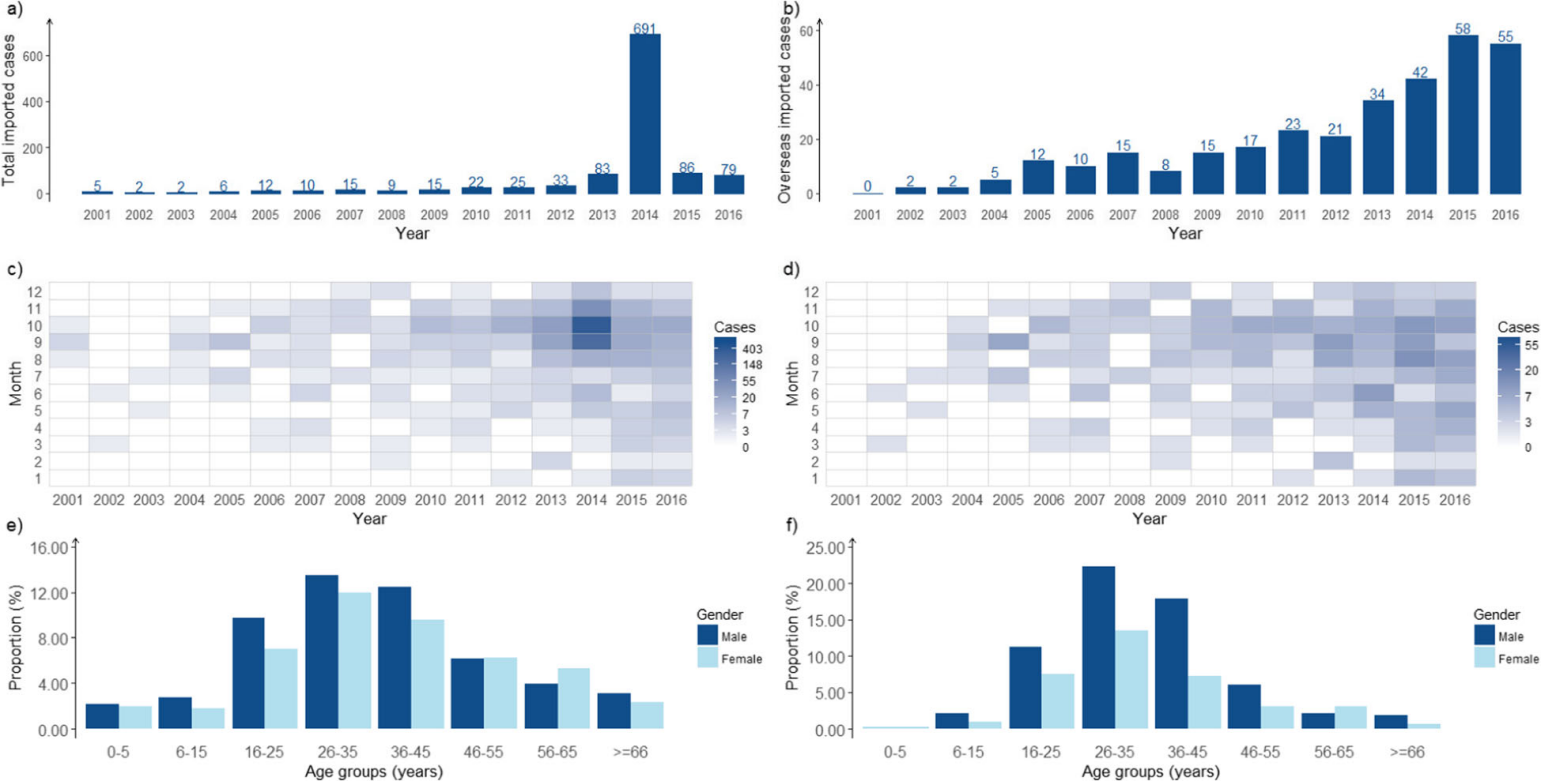
The epidemiological distribution of imported dengue cases in Guangzhou, 2001–2016. **a** Yearly distribution of the total number of imported cases; **b** Yearly distribution of the number of overseas imported cases; **c** Monthly distribution of the total number of imported cases; **d** Monthly distribution of the number of overseas imported cases; **e** Age group distribution by gender of the total number of imported cases; **f** Age group distribution by gender of the number of overseas imported cases

Between 2001 and 2016, a total of 1095 (2.58%) imported cases were reported to NIDRIS in Guangzhou, including 319 overseas imported cases and 776 domestic imported cases from other cities in china. The total number of imported cases peaked at 2014 with 691 cases reported, including 42 overseas imported cases and 649 domestic imported cases. The number of overseas imported cases showed a rising trend, from 0 in 2001 to 55 in 2016, with a peak number of 58 in 2015 (Fig. 3a and b).

In terms of seasonality, no imported case was identified in January before 2012. After 2012, imported cases were observed across all 12 months every year, with the majority of imported cases occurred from March to December, in which period a total of 1080 imported cases (98.63%) and 304 overseas imported cases were reported (95.30%) (Fig. 3c and d).

The overall male-to-female case ratio of total imported cases and overseas imported cases was 1.16 (589:506) and 1.75 (203:116) respectively. The characteristic of age distribution shows no difference between total imported cases and overseas imported cases. Those aged from 26 to 45 constituted the largest proportion of overseas imported cases (60.82%) and total imported cases (47.58%) (Fig. 3e and f).

### Virus isolation

**Fig. 4:**
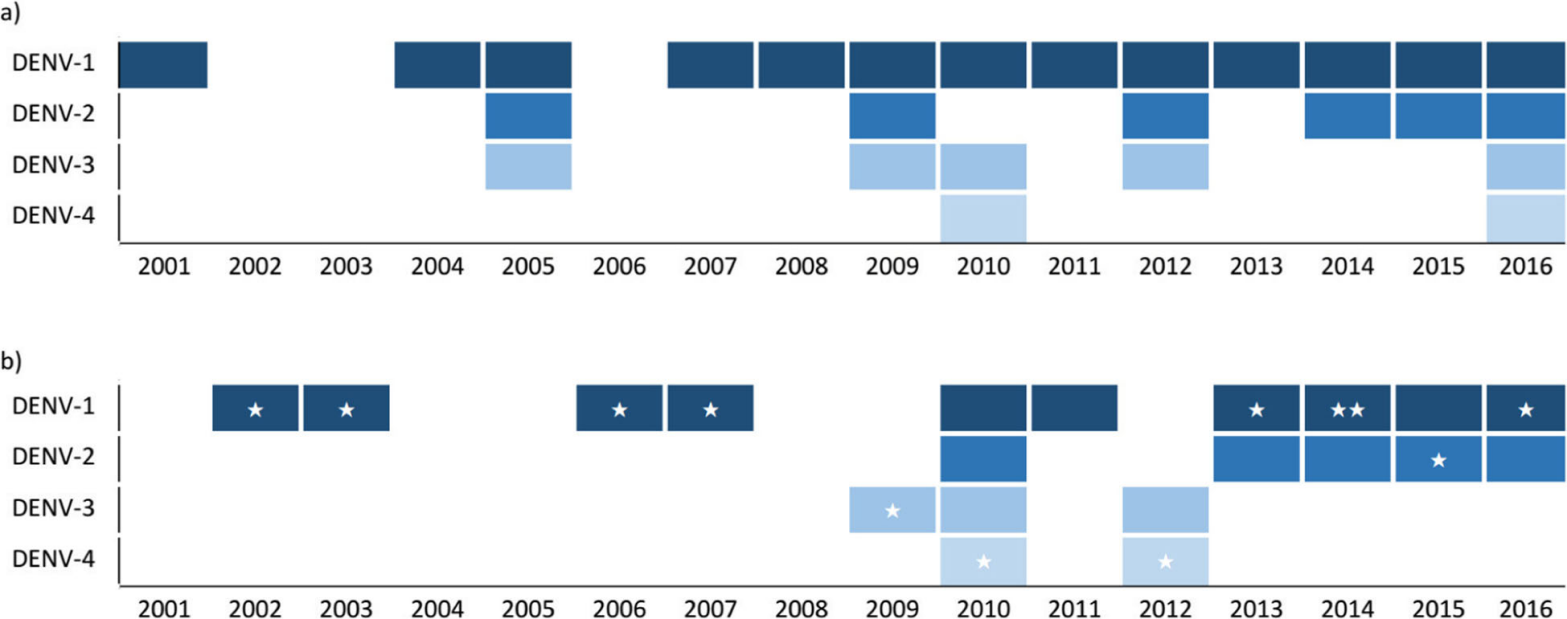
The yearly distribution of dengue virus strains in Guangzhou, 2001–2016. **a** The yearly distribution of dengue virus strains of imported cases; **b** The yearly distribution of dengue virus strains of indigenous cases, with star denoting the main transmission serotype

A total of 248 DENV strains were isolated in GZCDC from 2001 to 2016, including 193 (77.82%) strains from indigenous cases and 55 (22.17%) strains from imported cases. All the four DENV serotypes (DENV-1 to 4) were detected in Guangzhou from both indigenous cases and imported cases. Among indigenous cases, 152 DENV-1 strains were isolated and accounted for the largest proportion of 78.8%. DENV-1was the main epidemic strain in 2002–2003, 2013–2014, and 2016. DENV-2 was the main epidemic strain in 2015, DENV-4 in 2010 and 2012 and DENV-3 in 2009 (Fig. 4b). DENV-1 was also the main serotype of imported cases in Guangzhou and was detected in 13 (81.25%) years of the total 16 years in our study period. From 2001 to 2016, DENV-2 and DENV-3 were detected in 6 years and 5 years respectively (Fig. 4a).

### Vector surveillance

**Fig. 5:**
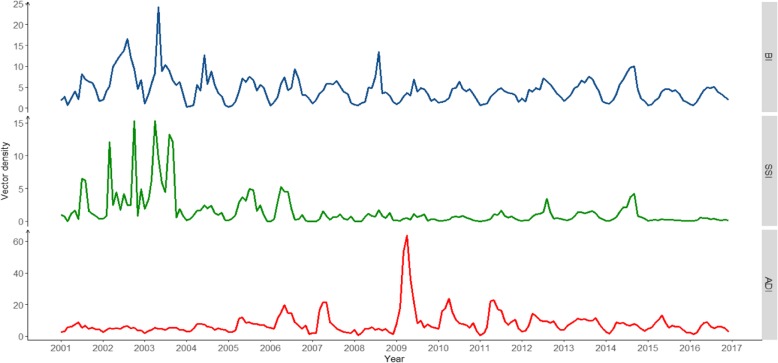
Time series of vector density for BI, SSI, and ADI in Guangzhou, 2001–2016

Vector surveillance during the study period revealed that the predominant mosquito species in Guangzhou was *Culex pipens pallens. Aedes albopictus* ranked second and was the only vector capable of transmitting dengue viruses in Guangzhou. No *Aedes aegypti* was identified. Vector density showed strong seasonal periodicity in Guangzhou, increasing around March and decreasing around October. The monthly median of BI, SSI and ADI was 4.41 (IQR: 2.05–5.76), 0.67 (IQR: 0.22–1.51), and5.93 (IQR: 4.17–8.70) respectively (Fig. 5).

## Discussion

Our study presents the longitudinal surveillance data for 16 years from 2001 to 2016 in Guangzhou city, the most important dengue epicenter in mainland China, and characterized the epidemiology of indigenous cases and imported cases, virus isolation and vector surveillance. We found that the overseas imported cases have increased over the past 16 years and there have been significant outbreaks of local transmission, especially dramatic in 2014.

During the study period, the geographic extent of reported dengue cases expanded continuously. Although the reported number of indigenous cases from 2006 to 2010 was lower than that in 2001–2005, an additional 23 communities were newly affected by indigenous cases in 2006–2010. A total of 120 communities were affected since 2001. The number of affected communities continued to climbing during the period 2011–2016 and the reported cases increased sharply. The central areas of the City with high population density had increasing risk of dengue infection over the three time periods. The large outbreaks in Guangzhou also result in exportation of dengue cases to other provinces in mainland China.

In our study, we observed that the incidence of indigenous cases increased with age. Another study showed that the middle age population had the highest incidence rate in Guangzhou [6]. But our study result showed that the incidence rate in age group below 25 years old and above 66 years old were high than age group between 26 and 65. The largest proportion of indigenous cases occurred in the population with the occupation of housework and the retired. This is different from endemic areas such as Southeast Asia where the higher incidence appeared in children or younger adults. A plausible explanation is that housework and the retired people in Guangzhou are more likely to spend their time on outdoor activities during 9a.m-11a.m and 4 pm–6 pm, which are consistent with the riskiest time for *Aedes albopictus* to bite people outside the door, whose ecological habitats are both outdoor and indoor, not the same as *Aedes aegypti* mostly active around residential environment. This phenomenon is not the same as the explanation in the south China [8].

As for imported dengue, the largest proportion of total imported cases occur in middle aged individuals and in males. A possible explanation is that these populations are more likely to travel abroad due to commence or tourism, thus having higher risk of exposure to dengue out of Guangzhou [4]. Actually, the dengue situation in Southeast Asia countries like Thailand, Malysia, Phillppine, and Singapore indicated increased dengue severity in the past three decades [9]. Other than overseas imported cases, domestic imported cases were imported from the adjacent areas like Foshan, Zhongshan, especially in 2014 record-breaking outbreak. Actually, the dengue viruses in those adjacent areas were also imported from oversea travel and then spread to nearby city such as Guangzhou.

In our study, all four DENV serotypes were found in Guangzhou city from indigenous cases. DENV-1 was the main transmission serotype which had two-year circulation in periods 2002–2003, 2006–2007, and 2013–2014; in 2016 it was also the main serotype in circulation. DENV-2 was the main transmitted serotype in 2015, and was also isolated in 2010, 2013–2014 and 2016, alhough no large outbreak was triggered by DENV-2. DENV-3 was isolated in 2009, 2010 and 2012 in confined areas without causing outbreaks. DENV-4 from indigenous cases was the main strain in 2010 and 2012, when the epidemics were triggered by oversea imported cases [5].. Among the imported cases, DENV-1 was the main serotype, followed by DENV-2, DENV-3 and DENV-4. The strong correlation of DENV serotypes that isolated from both indigenous cases and imported cases indicated that Guangzhou was the dengue imported area [5, 10]. There was a co-circulation of DENV-1 and DENV-2 in Guangzhou from 2013 to 2016 annually, but DENV-1 was the main serotype leading to major outbreak in 2002, 2006, 2013 and 2014. With the genotype diversity, DENV-1 genotype I maybe locally circulated, and genotype IV introduced in 2013 and resulted the record-breaking outbreak in 2014 [11].

*Aedes albopictus* is the only vector for dengue transmission in Guangzhou, It usually deemed as a mild vector that can’t lead to severe dengue outbreak. However, the record-breaking outbreak in 2014 shows it possesses the potential for a severe dengue outbreaks. There is still no *Aedes aegypti* be identified in Guangzhou in 2014. The habit and life circle of *Adede albopitus* plays critical role in the dengue epidemic in Guangzhou. Recent studies indicate that the propagation efficiency of *Aedes albopictus* for DENV transmission is as high as that of *Aedes aegypti*, and *Aedes albopictus* infected with DENV show higher concentrations of DENV RNA in abdominal tissues compared to *Aedes aegypti* [12]. Because *Aedes albopictus* can live both inside and outside of the resident house, and its risky time of biting is highly consistent whit the time when domestic workers and retirees carry out their outdoor activities. This prompt the speculation that infection occur mainly from outdoor places such as parks and other public places, not the same areas where dengue is transmitted by *Aedes aegypti* in places such as inside or around houses. The vector density from 2011 to 2016 was not significantly higher than in the period of 2001–2005 and 2006–2010, however, as more indigenous cases were reported. This may reflect that the vector capacity of *Aedes albopictus* related with biting rate, probability of vector survival, extrinsic incubation period and vector competence, all may play important role in virus transmission [13].

Most part of mainland China was characterized as an imported dengue area [4, 14]. Overseas imported cases after 2010 were significant higher than before in our study, similar to local transmission. Due to their widespread distribution, DENV-2, DENV-3, and DENV-4 still can cause imported epidemics in Guangzhou. However, there are 72 communities affected by DENV-1 and reported indigenous cases of DENV-1 during all the three periods of 2001–2005, 2006–2010, and 2010–2016, most of which occurred in central Guangzhou with highest population density. DENV-1 continues to appear every year in some communities, indicating that DENV-1 had a strong tendency towards local circulation [11].

There exist some limitations in our study. First, all the data analyzed was extracted from the surveillance system before 2005 was reported by telephone or email, and after 2005 was reported by the online system. As the reporting methods were different between these two stages, the data quality may be also different. Second, the number of reported cases may be lower than the actual infections due to inapparent infection and under-diagnosis and reporting [15]. Third, some cases are reported with no disease severity and serosurvey data, so the full spectrum of dengue in south China may still be worthy of further study to understand the full disease spectrum. Finally, a small number of imported cases and early-stage indigenous cases might be failed to be diagnosed in time by some medical institutions which lack of awareness of dengue diagnosis, resulting in missed timing of RT-PCR test and virus isolation. And We did not conduct virus isolation for most cases, we mainly perform virus isolation for the first and early cases of each outbreak and the proportion of virus isolation of late-stage cases was relatively low. This will affect the representativeness of our virus isolation and sequence analysis.

The climate and imported cases are the most important factors leading to dengue outbreaks in Guangzhou [16]. As the third largest city in mainland China, with exchange of commerce and population is continuing to increase, the risk of dengue transmission continues to raise in Guangzhou. This could create a further threat to other areas in mainland China. Furthermore, the population density in Guangzhou also continue to grow with expansion peri-urban areas. Even now, further researches are needed to focus on potential factors that lead to dengue transmission, potential treatments and new vector control strategies with the ultimate objective of conquering dengue [17].

## Conclusion

In this study, we found that the number of indigenous cases was on the rise, the frequency of large-scale outbreaks was increasing and the affected area of dengue outbreak also expanded sharply geographically 2001–2016. Furthermore, all four dengue virus serotypes were identified in indigenous cases and DENV-1 continuously detected in resent years, Guangzhou has become the dengue epicenter in mainland China. Control strategies for dengue should be adjusted to the epidemiological characteristics above and intensive study need to be conducted to explore the factors that driving the rapid increase of dengue.

## Data Availability

The data that support the findings of this study are available from the National Notifiable Infectious Disease Reporting Information System (NIDRIS) that managed by the National Health and Family Planning Commission of China. Due to the information security policy of the National Health and Family Planning Commission of China, the datasets are not publicly available. Data are however available from the authors upon reasonable request and with permission of the National Health and Family Planning Commission of China.

## Abbreviations

ADE: Antibody dependent enhancement
ADI: The Adult Mosquito Density Index
BI: The Breteau Index
DENV: Dengue virus
DHF: Dengue hemorrhagic fever
DSS: Dengue shock syndrome
GZCDC: The Guangzhou Center for Disease Control and Prevention
NIDRIS: The National Notifiable Infectious Disease Reporting Information System
qRT-PCR: Real-time fluorescent quantitative reverse transcription-PCR
SSI: The Standard Space Index

## References

[1] Bhatt S, Gething PW, Brady OJ, Messina JP, Farlow AW, Moyes CL (2013). The global distribution and burden of dengue. Nature.

[2] Guzman MG, Harris E (2015). Dengue. Lancet.

[3] Qiu FX, Gubler DJ, Liu JC, Chen QQ (1993). Dengue in China: a clinical review. Bull World Health Organ.

[4] Lai S, Huang Z, Zhou H, Anders KL, Perkins TA, Yin W (2015). The changing epidemiology of dengue in China, 1990-2014: a descriptive analysis of 25 years of nationwide surveillance data. BMC Med.

[5] Jing QL, Yang ZC, Luo L, Xiao XC, Di B, He P (2012). Emergence of dengue virus 4 genotype II in Guangzhou, China, 2010: survey and molecular epidemiology of one community outbreak. BMC Infect Dis.

[6] Luo L, Liang HY, Hu YS, Liu WJ, Wang YL, Jing QL (2012). Epidemiological, virological, and entomological characteristics of dengue from 1978 to 2009 in Guangzhou. China J Vector Ecol.

[7] Team RC (2017). R: a language and environment for statistical computing.

[8] Guo RN, Lin JY, Li LH, Ke CW, He JF, Zhong HJ (2014). The prevalence and endemic nature of dengue infections in Guangdong, South China: an epidemiological, serological, and etiological study from 2005-2011. PLoS One.

[9] Lefevre A. Thailand suffers worst dengue epidemic in more than 20 years. Thomson Reuters Foundation. 2013.

[10] Liang H, Luo L, Yang Z, Di B, Bai Z, He P (2013). Re-emergence of dengue virus type 3 in Canton, China, 2009-2010, associated with multiple introductions through different geographical routes. PLoS One.

[11] Jiang LY, Jing QL, Liu Y, Cao YM, Su WZ, Biao D (2017). Molecular characterization and genotype shift of dengue virus strains between 2001 and 2014 in Guangzhou. Epidemiol Infect.

[12] Whitehorn J, Kien DT, Nguyen NM, Nguyen HL, Kyrylos PP, Carrington LB (2015). Comparative susceptibility of Aedes albopictus and Aedes aegypti to dengue virus infection following human viremic blood-feeding: implications for public health. J Infect Dis.

[13] Lambrechts L, Paaijmans KP, Fansiri T, Carrington LB, Kramer LD, Thomas MB (2011). Impact of daily temperature fluctuations on dengue virus transmission by Aedes aegypti. Proc Natl Acad Sci U S A.

[14] Sang S, Chen B, Wu H, Yang Z, Di B, Wang L (2015). Dengue is still an imported disease in China: a case study in Guangzhou. Infect Genet Evol.

[15] Wang T, Wang M, Shu B, Chen XQ, Luo L, Wang JY (2015). Evaluation of inapparent dengue infections during an outbreak in southern China. PLoS Negl Trop Dis.

[16] Cheng Q, Jing Q, Spear RC, Marshall JM, Yang Z, Gong P (2016). Climate and the timing of imported cases as determinants of the dengue outbreak in Guangzhou, 2014: evidence from a mathematical model. PLoS Negl Trop Dis.

[17] Katzelnick LC, Coloma J, Harris E (2017). Dengue: knowledge gaps, unmet needs, and research priorities. Lancet Infect Dis.

